# Tipping point of plant functional traits of *Leymus chinensis* to nitrogen addition in a temperate grassland

**DOI:** 10.3389/fpls.2022.982478

**Published:** 2022-08-17

**Authors:** Guojiao Yang, Zijia Zhang, Guangming Zhang, Qianguang Liu, Peiming Zheng, Renqing Wang

**Affiliations:** ^1^School of Life Sciences, Institute of Ecology and Biodiversity, Shandong University, Qingdao, China; ^2^Key Laboratory of Agro-Forestry Environmental Processes and Ecological Regulation of Hainan Province, Hainan University, Haikou, China; ^3^Hainan Ecological Environment Monitoring Center, Haikou, China; ^4^Department of Pharmaceutical Science, Changzhi Medical College, Changzhi, China; ^5^Shandong Provincial Engineering and Technology Research Center for Vegetation Ecology, Shandong University, Qingdao, China; ^6^Qingdao Forest Ecology Research Station of National Forestry and Grassland Administration, Shandong University, Qingdao, China

**Keywords:** nitrogen deposition, threshold, functional traits, productivity, steppe

## Abstract

It has widely been documented that nitrogen (N) enrichment stimulates plant growth and modifies plant functional traits in the terrestrial ecosystem. However, it remains unclear whether there are critical transitions or tipping points for the response of plant growth or traits to N enrichment, and how these responses differ to different N forms. We chose the native, perennial clonal grass, *Leymus chinensis* in Inner Mongolia steppe, and conducted a field experiment, in which six N addition rates (0, 2, 5, 10, 20, and 50 g N m^–2^ year^–1^) and five N compound types [NH_4_NO_3_, (NH_4_)_2_SO_4_, NH_4_HCO_3_, CO(NH_2_)_2_, slow-release CO(NH_2_)_2_] are considered. Here, we found that the different N compound types had no significant effect on the growth of *L. chinensis*. N addition rate significantly increased plant aboveground biomass and leaf nitrogen concentration, whereas decreased leaf dry matter content. The tipping point for N-induced aboveground biomass increase was at 10 g N m^–2^ year^–1^, and the changes in functional traits were at N addition rates of 20 g N m^–2^ year^–1^. Our findings suggested that the responses of aboveground biomass and functional traits to N addition were asymmetric, in which responses in aboveground biomass were more sensitive than that in functional traits. The differential sensitivity of aboveground biomass and functional traits of *L. chinensis* occurred to N deposition highlights the importance of functional traits in mediating ecosystem functioning in the face of N deposition, regardless of which chemical forms dominate in the deposited N.

## Introduction

Nitrogen (N) is a major limiting resource for plant growth in diverse natural ecosystems (Vitousek and Howarth, 1991; Elser et al., 2007) and plays a crucial role in photosynthesis and all enzymatic activities (Reich et al., 2004). Human activities such as fossil fuel combustion and fertilizer application have dramatically increased reactive N being deposited into many terrestrial ecosystems (Galloway et al., 2008). There likely exist ecosystem-specific thresholds for N enrichment, beyond which ecosystem start to transit substantially, such as the critical threshold for N-induced species loss to mature Eurasian grasslands is 1.75 g N m^–2^ year^–1^ (Bai et al., 2010). An ecological threshold or tipping point is the point at which there is an abrupt change in an ecosystem quality, property, or phenomenon (Groffman et al., 2006). Identifying ecological thresholds in response to climate change such as N deposition is important to guide ecosystem management and maintain ecological services (Rockström et al., 2009).

Plant functional traits reflect plants’ capacities for resource capture and adaptations to environmental changes (Wright et al., 2004). As plant economic spectrum predicts, species with the traits capable of acquiring resource rapidly (e.g., high N concentration, specific leaf area, and photosynthetic rate) would compete for resource more strongly under N enrichment (Wright et al., 2004; La Pierre and Smith, 2015). Recent studies have revealed inconsistent responses of plant functional traits to nutrient addition, while some studies showed that N addition enhanced leaf N concentration, chlorophyll concentration and specific leaf area, thus leading to a marked increase in photosynthetic rates (Tatarko and Knops, 2018; Zheng et al., 2018; Sun et al., 2022). Others found no effect of N addition on leaf mass area, area-based concentrations of foliar N and chlorophyll concentrations (Hu and Wan, 2019). One possible explanation for the inconsistency among these studies is that the response of plant functional traits to N enrichment may be non-linear (Zhang et al., 2019; Song et al., 2022), such that plant functional traits may not necessarily show a significant change until a threshold N level is reached. Nevertheless, it remains unclear whether there is tipping point for the effect of N inputs on plant traits. Moreover, plasticity in plant traits is predicted to play a key role in affecting plant production. For example, the study conducted in the central French alpine grassland found that the productivity was positively associated with fertilization-induced increases in plant height and leaf area (Lavorel and Grigulis, 2012). Another study conducted in an Chinese alpine steppe found that increasing leaf N content and leaf area under N addition significantly promoted plant community productivity (Peng et al., 2017; Zhang et al., 2021). However, to date, very few studies have considered whether plant traits can be associated with the response of plant production to increased N inputs.

In addition to plant functional traits, N enrichment could influence plant growth and productivity by changing soil environmental conditions such as soil N availability and acidification (Bobbink et al., 1998). Increasing soil N availability was the key direct factor driving the plant growth under N addition (Tao et al., 2022). In addition, N deposition can result in soil acidification both directly as a result of acid deposition (nitric acid) and indirectly through processes and reactions in soil and water (Stevens et al., 2010), which results in toxicity through mobilization of metals, leaching of base cations and changing the balance between nitrogenous compounds, and hence causes cascading effects on plant growth (Roem et al., 2002; Stevens et al., 2010).

In this study, we investigated the plant functional traits in a multi-level (0, 2, 5, 10, 20, and 50 g N m^–2^ year^–1^) N addition experiment in a temperate grassland in Inner Mongolia, China, to test whether there will be a N threshold for plant functional traits in response to N deposition. The temperate grassland in our study site is dominated by a native perennial rhizomatous grass *Leymus chinensis*, which is palatable for grazing animals and has high forage value and drought tolerance (Bai et al., 2004; Huang et al., 2015). Here, *L. chinensis* was chosen as a model plant, we hypothesized that (a) when the N addition level reaches a certain level, aboveground biomass and plant functional traits will cross the critical threshold and show dramatic response, and the threshold will be at similar N addition rate for aboveground biomass and traits, (b) changes in traits and soil conditions will together drive the response of plant production to increasing N addition.

## Materials and methods

### Study site

The experiment was conducted at the Erguna Forest-Steppe Ecotone Research Station (N50^°^10’46.1′′, E119^°^22’56.4′′), Institute of Applied Ecology, the Chinese Academy of Sciences. The grassland had been used for forage harvest before 2013. The long-term mean annual precipitation of the site is 363 mm and the mean annual temperature is -2.45°C (1957–2016). The soil is classified as chernozem according to the Food and Agricultural Organization of the United Nations classification. The dominant species in this ecosystem is *Leymus chinensis*, which makes up > 45.6% of the total aboveground biomass.

### Experimental design

The N addition experiment was started in 2014, with a randomized complete block design to study the effects of rates and types [NH_4_NO_3_, (NH_4_)_2_SO_4_, NH_4_HCO_3_, CO(NH_2_)_2_, slow-release CO(NH_2_)_2_] of N addition on plant biomass and leaf functional traits. There were six N addition rates (i.e., 0, 2, 5, 10, 20, and 50 g N m^–2^ year^–1^), and five types of N compounds. Each of the 30 treatments had eight replications, leading to 240 treatment plots in total. The area of each plot was 10 m × 10 m. Nitrogen fertilizers were added annually since 2014, in late May. Fertilizers were mixed with sand (because of the low amount of added fertilizer at low addition rates) and added uniformly by hand. Sand was sieved through less than 2 mm in size, washed in water, and then heated at nearly 250°C for 60 min in an iron pan. To avoid potentially confounding effects, all plots received the same amount of sand (0.5 kg per plot).

### Field sampling and measurement

At each plot, 15 healthy and mature plant individuals were randomly selected from different locations between 1st and 5th August, 2015. These plant individuals were clipped at the ground level and immediately placed in a portable cooler. In the laboratory, all samples were soaked in water for 6 h to ensure full rehydration. Each leaf was then cut from the stem and gently dried with tissue paper before measurement. Water-saturated leaf mass was weighed, scanned the leaf with a scanner (Canon LiDE120), and determined the leaf area by the ImageJ software. All leaves were then dried for 48 h at 65^°^C and then weighed. The leaf dry matter content (LDMC), specific leaf area (SLA), and stem: leaf ratio were calculated. After being ground in a ball mill (Retsch MM 400; Retsch, Haan, Germany), the leaf total N concentration was analyzed on an elemental analyzer (Vario MACRO cube, Elementar Analysensysteme, Germany). The grinded leaf samples (50 mg) were digested with H_2_SO_4_–H_2_O_2_ (Bennett et al., 2002), and measured colorimetrically for leaf P concentration at 880 nm after reaction with molybdenum blue. N: P ratio was reported as mass ratios.

Aboveground biomass of *L. chinensis* was sampled between August 10th and 20th by clipping plants at the soil surface in a 1 m × 1 m quadrat, which was randomly placed in each plot without a spatial overlap of quadrats among different years and at least 50 cm inside the border of each plot to avoid edge effects. All plant materials were oven-dried at 65°C for 48 h and weighed. After clipping above-ground biomass, three soil cores (0–5 cm depth and 50 cm apart) were collected using a 7 cm diameter soil auger adjacent to each aboveground plant sample plot and mixed *in situ* into one composite sample. Fresh soil samples were sieved through a 2-mm sieve to remove visible roots, plant residues, and stones, and taken to the laboratory for analysis of soil water content, soil inorganic N and pH. Fresh soil was extracted by 2 M KCl solution, 10 g of soil was extracted in 50 mL of 2 M KCl solution, and then inorganic N concentrations were analyzed with a FLAstar 5000 Analyzer (Foss Tecator, Hillerød, Denmark). Subsamples were air−dried and analyzed soil pH using a pH meter (Thermo Fisher Scientific, America).

### Statistical analysis

Linear mixed effect model analysis of variance was performed using the lme function from the nlme package with N addition rate and N compounds type as fixed factors and block as a random factor. Given N compounds showed no significant effect on plant biomass and functional traits (Table 1), we pooled data from different N compounds under a certain N addition rate to simplify our analysis and focus on the effect of N addition rate. We used Duncan’s test to evaluate aboveground biomass, SLA, LDMC, stem: leaf ratio, leaf N concentration, P concentration, leaf N: P ratio of *Leymus chinensis*, and SIN, soil pH and SWC under varying N addition rates. To examine how aboveground biomass of *Leymus chinensis* was correlated with SIN or soil pH across different N addition rate, a linear regression model was performed. All analyses were conducted using R version 4.0.2 (R Development Core Team, 2020).

**TABLE 1:** Results of mixed model analysis of variance for aboveground biomass, SLA (specific leaf area), LDMC (leaf dry matter content), stem: leaf ratio, leaf N concentration, leaf P concentration, leaf N: P ratio of *Leymus chinensis*, and soil inorganic nitrogen content (SIN), soil pH, soil water content (SWC).

	N addition rate	N compounds type	N addition rate × N compounds type
Aboveground biomass	55.64***	0.39^ns^	1.74^ns^
SPAD	14.22**	1.06^ns^	0.45^ns^
SLA	16.38**	0.79^ns^	0.64^ns^
LDMC	5.75*	0.43^ns^	2.27^ns^
Stem: leaf ratio	0.19^ns^	1.56^ns^	1.17^ns^
Leaf N content	30.84***	2.11^ns^	0.49^ns^
Leaf P content	0.03^ns^	0.53^ns^	0.28^ns^
Leaf N: P ratio	13.83***	2.25^ns^	2.53^ns^
SIN	136.19***	11.32***	2.35^ns^
Soil pH	54.32***	6.4***	0.405^ns^
SWC	6.62*	0.52^ns^	3.19*

N addition rate and N compounds type were used as fixed factors and block as a random factor. The F-values were shown. Asterisks denote significant levels: ns, *P* > 0.05; *P ≤ 0.05; **P ≤ 0.01; and ***P ≤ 0.001, respectively.

## Results

### Effects of N addition on plant functional traits of *Leymus chinensis*

**FIGURE 1:**
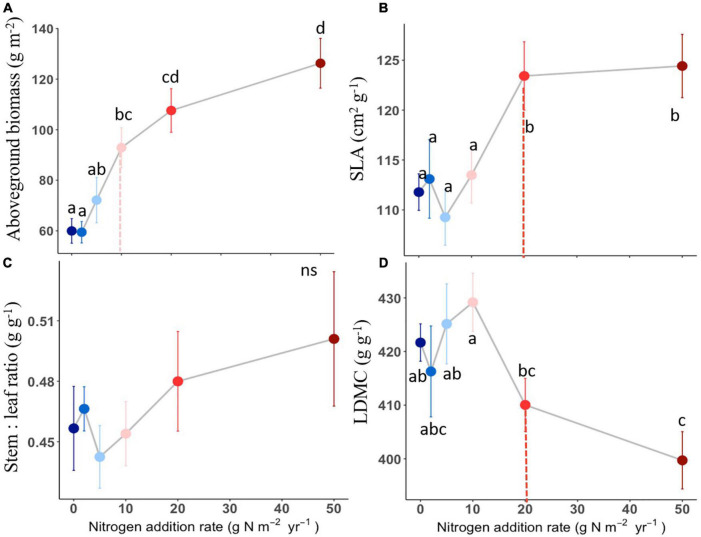
Effects of N addition on plant biomass and leaf traits of *L. chinensis*. Trait abbreviations are aboveground biomass **(A)**, SLA (specific leaf area) **(B)**, LDMC (leaf dry matter content) **(C)** and stem: leaf ratio **(D)**. Different lower-case letters indicate significant differences (*P* < 0.05) among treatments, and ns indicates non-significant (*P* > 0.05). The dashed line in each panel indicate for tipping points. The data shown are the means with 40 replications ± standard error.

**FIGURE 2:**
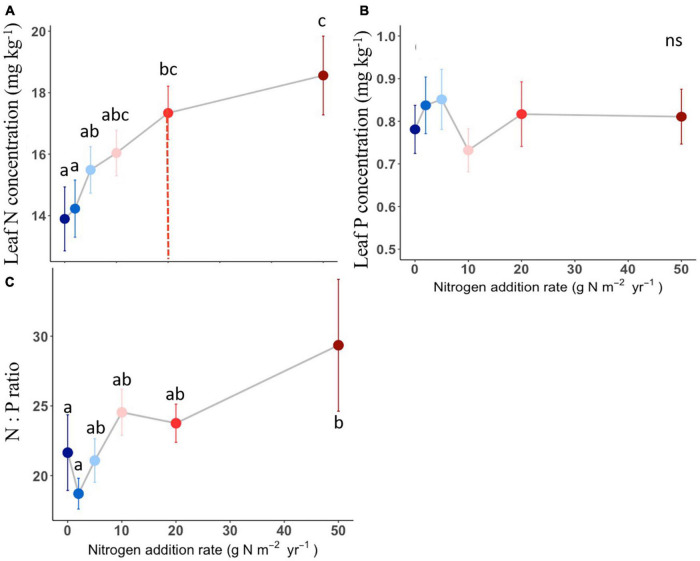
Effects of N addition on leaf stoichiometry of *L. chinensis*. Trait abbreviations are leaf N concentration **(A)**, leaf P concentration **(B)**, leaf N: P mass ratio **(C)**. Different lower-case letters indicate significant differences (*P* < 0.05) between treatments, and ns indicates non-significant (*P* > 0.05). The dashed line in each panel indicate for tipping points. The data shown are the means with 40 replications ± standard error.

The compound types of added N had no significant effects on aboveground biomass, SLA, LDMC, stem: leaf ratio, leaf N content, leaf P content, leaf N: P ratio of *L. chinensis* (Table 1, all *P* > 0.05). By contrast, N addition rates showed dramatic effects on aboveground biomass, leaf morphology and stoichiometry. Aboveground biomass was not different among the control and N addition treatments up to 5 g N m^–2^ year^–1^ but significantly increased at the three highest N addition levels (10, 20, and 50 g N m^–2^ year^–1^; Figure 1A). N addition rate increased SLA and leaf N concentration whereas decreased LDMC (Figures 1B,D, 2A), with the tipping point of N-induced changes being at the rate of 20 g N m^–2^ year^–1^. The stem: leaf ratio and leaf P concentration were not affected by N addition (Figures 1C, 2B).

### Effects of N addition on soil characteristics

**FIGURE 3:**
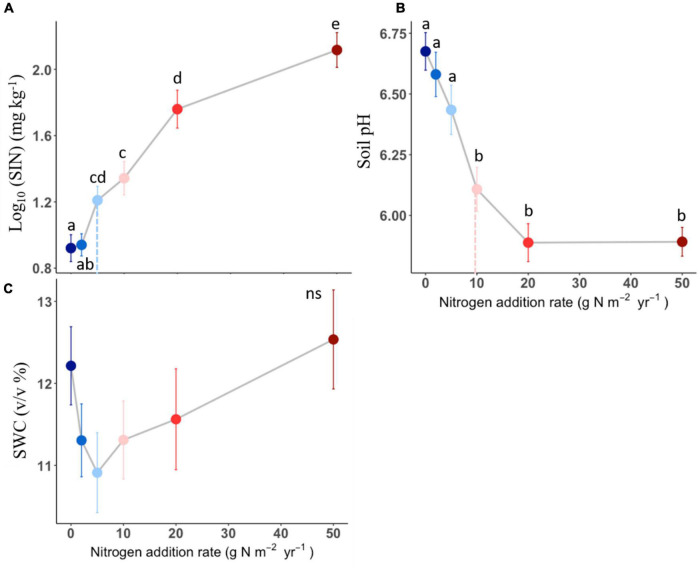
Effects of different N addition on soil inorganic nitrogen (SIN, log_10_-transformed, **A**), soil pH **(B)**, soil water content (SWC, **C**). Different lower-case letters indicate significant differences (*P* < 0.05) between treatments, and ns indicates non-significant (*P* > 0.05). The dashed line in each panel indicate for tipping points. The data shown are the means with 40 replications ± standard error.

Soil inorganic nitrogen increased with N addition once the N addition rate passed the 2 g N m^–2^ year^–1^ (Figure 3A). Soil pH in the plots receiving 10, 20, and 50 g N m^–2^ year^–1^ was lower than the one receiving N below 10 g N m^–2^ year^–1^ level (Figure 3B). However, there was no detectable difference in soil water content among N addition rates (Figure 3C). The effects of N addition on soil inorganic N and soil pH significantly different among N compounds (*P* < 0.05, Table 1 and Supplementary Figure 1), with the (NH_4_)_2_SO_4_ treatment showing stronger effect than other N compounds.

### Correlations between aboveground biomass and soil conditions

**FIGURE 4:**
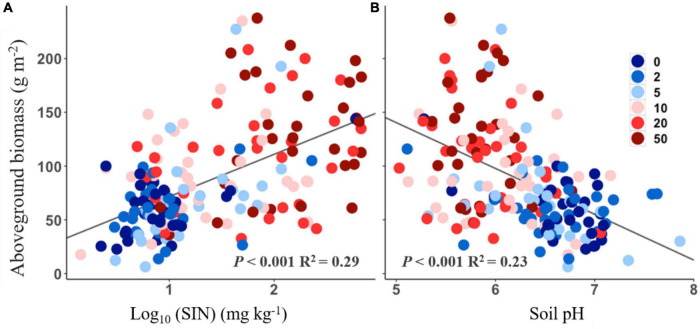
Relationships between aboveground biomass and soil inorganic nitrogen (SIN, log_10_-transformed, **A**) and soil pH **(B)**.

The aboveground biomass was positively correlated with soil inorganic N (Figure 4A, *R*^2^ = 0.29, *P* < 0.001), but negatively correlated with soil pH (Figure 4B, *R*^2^ = 0.23, *P* < 0.001). No significant relationship, however, was found between aboveground biomass and functional traits response to N addition (all *P* > 0.05).

## Discussion

### Non-linear response of aboveground biomass to N addition

Consistent with our hypothesis, we found that aboveground biomass of *L. chinensis* was not affected by low levels of N addition (i.e., < 5 g N m^–2^ year^–1^), but sharply increased between 10 and 50 g N m^–2^ year^–1^. These results suggested that plant growth in Inner Mongolia grassland was strongly limited by N and N addition could stimulate plant growth by relieving N limitation, which agreed with observations in Hautier et al. (2009). Moreover, another field experiment in Inner Mongolia at the typical steppe found that increase in N availability also stimulated community aboveground biomass, with the significant positive response occurred at the N addition rate of 1.75 g N m^–2^ year^–1^ by the fourth year (Bai et al., 2010). It is lower than that in our study where N threshold was ≥ 10 g N m^–2^ year^–1^. This may be mainly due to that our study have been performed just for 2 years, which has lower soil N availability. So the change of *L. chinensis* under long-term N addition should be investigated in future studies.

Nitrogen-induced changes in soil conditions could mediate aboveground biomass responses to the increasing N addition rate. Correlations between aboveground biomass and soil N availability vs. soil pH indicate that aboveground production is sensitive to changes in soil properties (Figure 4). First, the increase in soil N availability could contribute to plant growth (Gough et al., 2000), especially in our experiment. This because the model species *L. chinensis* is typical clonal plant. It has been reported that clonal plants have competitive advantages in acquiring soil nutrients and light, which cause their response to N addition to be more positive than non-clonal plants (Gough et al., 2012; Dickson et al., 2014). Second, the soil acidification under N addition could be another reason for the loss of aboveground production. For example, previous study found that decreased soil pH may lead to an increase in mortality of acid−sensitive plants (van den Berg et al., 2005), and induce decreases in plant productivity (Chen et al., 2013). Many studies have documented that soil acidification increases Al^3+^, which can be directly toxic to plants (van den Berg et al., 2005). However, a previous study showed that *L. chinensis* was positively correlated with the degree of acidification in a temperate steppe (Lan, 2014), which was in line with our results here. Both results suggested *L. chinensis* could be tolerant to acid conditions and its relative biomass could continue to increase with increasing N addition even though such a scenario soil acidification became severe (Yang et al., 2019).

### Non-linear response of plant functional traits to N addition

Contrary to our hypothesis, we did not find a similar threshold in response to N addition between plant biomass and functional traits. For example, the threshold for N-induced plant traits changes was at N addition rate of 20 g N m^–2^ year^–1^, higher than that of aboveground biomass (Figures 1, 2). It reflected that plant traits was relatively less sensitive than the aboveground biomass to N addition rate from the short-term N deposition. The distinct N threshold between the aboveground biomass and plant traits could be explained by the following two aspects. First, N is the limiting nutrient for productivity in the temperate steppe, and N enrichment could increase aboveground production (Bai et al., 2010). The enhanced plant growth induced by N addition could increase the intensity of light competition among plant species (DeMalach et al., 2017). Plants usually develop leaves with high SLA to enhance light capture (Freschet et al., 2015). Therefore, changes of SLA could reflect their adaptive capacities to low light conditions after the enhanced plant growth induced by N addition. Second, the response of traits to one global change driver may also be associated with other drivers. Given that water and N availability can co-limit plant growth in semiarid regions (Hooper and Johnson, 1999; Niu et al., 2010). It is expected that drought would restrain plant N uptake, thus the effects of changing precipitation regimes and global N enrichment will be interdependent (Shen et al., 2020). The long-term mean annual precipitation of the study site is 363 mm, and our study was conducted in a drought year (the mean annual precipitation is 148.2 mm). Previous study found that drought had a stronger effect on leaf traits than short-term N deposition, and the effect of drought on traits is opposite to that of N addition, for example drought had significant negative effects on the leaf N concentrations and net photosynthetic rate (Yu et al., 2019). Therefore, the threshold for N-induced plant traits changes was higher in this study.

High N addition rates increased SLA and leaf N content, decreases LDMC, which is consistent with many previous findings (La Pierre and Smith, 2015; Wang et al., 2016; Valliere et al., 2017; Zheng and Ma, 2018). For those traits, first, SLA indicates captured light resources on LA per unit leaf dry matter investment, which is closely related to plants’ light interception efficiency (Reich, 2014). Previous studies have shown that N addition increased SLA, possibly because it promoted growth and thus increased leaf photosynthesis, or reduced the availability of canopy light to increase the SLA (Zeng et al., 2009; Palmroth et al., 2014). Second, Leaf N is an integral component of the photosynthetic machinery, and changes in leaf N concentration may underpin the greater competitive capability of *L. chinensis* in the community under enhanced N input (Zheng et al., 2018). In line with our results, a recent work using 2683 observations showed that N addition enhanced leaf N concentration both on mass basis and area basis (Liang et al., 2020). LDMC is an index of conservatism in life history. We found that LDMC decreased under N addition, and low LDMC represent rapid nutrient acquisition, which is conducive to the growth of plants in a nutrient-rich environment (Wilson et al., 1999). Overall, elevated SLA and leaf N content, declined LDMC caused by N enrichment suggests that *L. chinensis* have a resource acquisitive strategy and such strategy become stronger under exogenous N input.

Here we targeted one single species under short-term N addition by focusing on traits of aboveground tissue, which raised few limitations that need to be addressed in future studies. First, it has been reported that clonal plants response more positively than non-clonal plants to N addition (Dickson et al., 2014). Therefore, further research is needed to explore how other species respond to N addition, whether the responses of aboveground biomass and functional traits to N addition were asymmetric for other species. Second, this study is based on a relatively short-term N-enrichment experiment. The response of functional traits and productivity may differ between short-term and long-term experiments. Therefore, long- term N addition experiments are needed. Thrid, root traits also play pivotal roles in resource acquisition, as plant economic spectrum predicts, species with the traits of high specific root length and low tissue density would more strongly compete for belowground resource (Weemstra et al., 2016; Erktan et al., 2018). Recognition of the different response between above- and below-ground traits could improve our understanding of plant growth to N addition. Thus we need explore further how the root traits would affect plant growth under N deposition scenarios.

## Conclusion

By conducting a multi-level of rates and chemical forms of N addition at the field condition in Chinese temperate grassland, here we show evidence to support that there indeed exist tipping points for plant biomass and functional traits in response to N addition. In particular, the tipping point for N-induced increase in *L. chinensis* aboveground biomass is at N addition rates of 10 g N m^–2^ year^–1^, while that critical points in functional traits are at 20 g N m^–2^ year^–1^, suggesting that the responses to N addition were more sensitive in aboveground biomass than that in plant functional traits. Our findings can help to understand to what extent and in which rate of N deposition will start to cause the transition of plant functional traits and biomass production. These insights certainly can be useful in forecasting changes in ecosystem service and maintaining ecosystem management under future N deposition scenarios.

## Data availability statement

The original contributions presented in the study are included in the article/Supplementary material, further inquiries can be directed to the corresponding authors.

## Author contributions

GY, ZZ, and GZ collected the data. GY, ZZ, PZ, and RW developed the research questions. GY and QL analyzed the data. GY wrote the draft with contributions and input from all authors.
