# Supplementary material for: Tipping point of plant functional traits of Leymus chinensis to nitrogen addition in a temperate grassland
Source: Front Plant Sci. 2022 Aug 17;13:982478. doi: 10.3389/fpls.2022.982478 (PMC9428514; doi:10.3389/fpls.2022.982478)
Supplement: Supplementary file 1 [file Table_1.DOCX]

**Figure S1** Effects of N addition rate and N compounds type on log_10_ (SIN) (soil inorganic nitrogen content, A), soil pH (B). The data shown are the means with 8 replications ± SE.


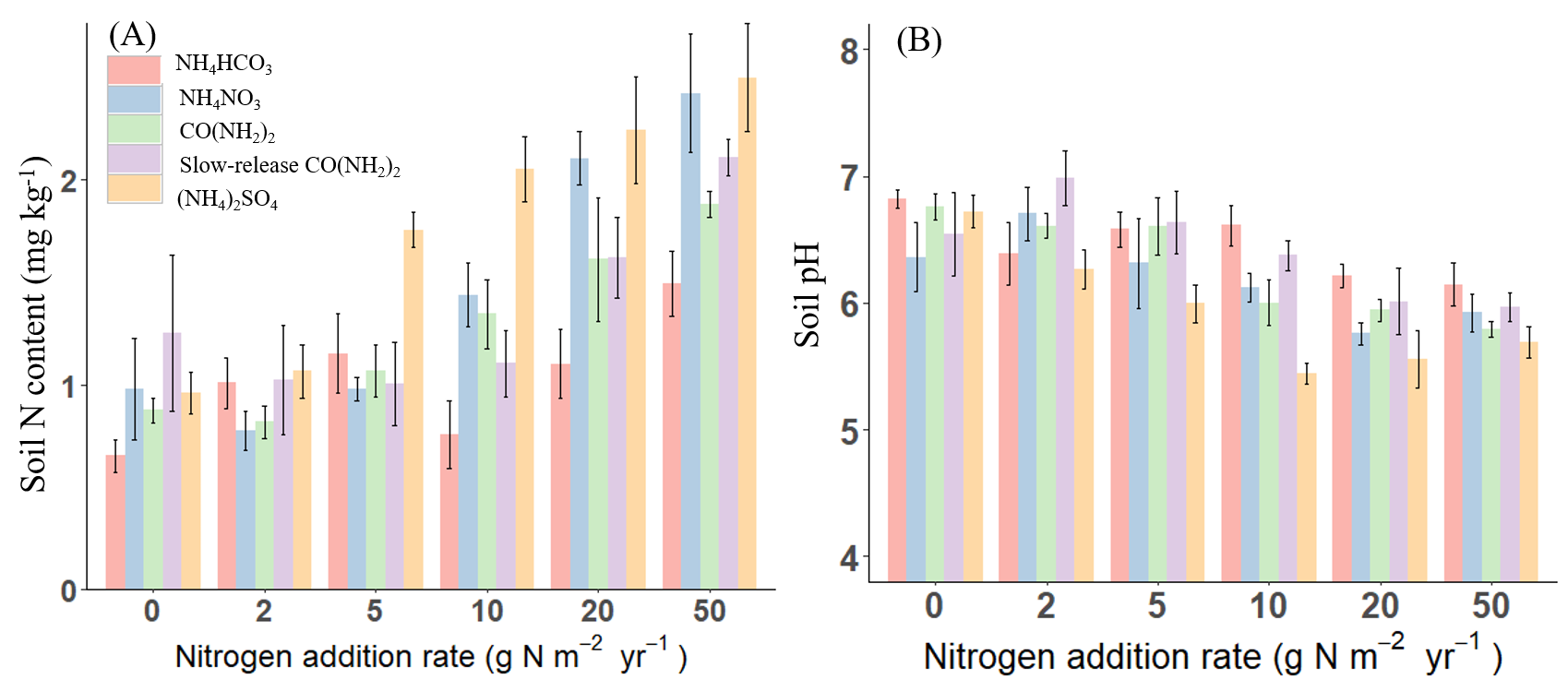


**Figure S1**
